# Interest in peer support persons among patients experiencing early pregnancy loss

**DOI:** 10.1186/s12884-023-05816-x

**Published:** 2023-07-11

**Authors:** Carmen Conroy, Tanya Jain, Sheila K. Mody

**Affiliations:** 1 School of Medicine, University of California at San Diego, San Diego, CA USA; 2 Division of Complex Family Planning, Department of Obstetrics, Gynecology, and Reproductive Sciences, University of California at San Diego, 9300 Campus Point Dr. MC 7433, La Jolla, 92037 San Diego, CA USA

**Keywords:** Miscarriage, Early pregnancy loss, Self-compassion, Peer support

## Abstract

**Background:**

Limited data exist regarding the type of support patients need when experiencing early pregnancy loss (EPL). The objective of this study is to explore how patients emotionally cope with EPL and to assess if there is interest in a peer EPL support program with a self-compassion component.

**Methods:**

We conducted semi-structured interviews with patients who experienced EPL in the past two years. We evaluated the kinds of support that patients felt were most helpful, interest in a possible peer EPL support person, and suggestions for the creation of such a program. Content analysis was utilized to analyze the data and identify themes.

**Results:**

Twenty-one individuals participated in the study. Approximately 52.3% (n = 11) of interviewees reported expectant management of their EPL, 23.8% (n = 5) reported medication management, and 23.8% (n = 5) reported undergoing dilation and curettage. We identified five themes: (1) therapy and in-person support groups are helpful when experiencing EPL, but are sometimes inaccessible; (2) social media support groups are initially advantageous for creating a sense of solidarity, but in the long term can be triggering; (3) support from a peer who has also experienced EPL is uniquely valuable; (4) developing self-compassion is important in emotionally coping with EPL; and (5) there is a demand for emotional and informational support following EPL.

**Conclusions:**

Given the unique support participants identified receiving from a peer with shared lived experience, there is interest in a peer EPL support program with a self-compassion component for emotional and informational support following EPL.

**Supplementary Information:**

The online version contains supplementary material available at 10.1186/s12884-023-05816-x.

## Introduction

Approximately 6.5 million early pregnancy losses (EPLs) occur every year in the United States, and it is estimated that 10–25% of women report having an EPL [1]. Management of EPL can include a dilation and curettage (D&C) procedure, or medication management, which involves taking a medication and passing the pregnancy at home. EPL can result in possible psychological sequalae, including the increased risk of developing anxiety, depression, post-traumatic stress disorder, and suicidal ideations [1, 2]; however, limited data exist to elucidate which factors might help attenuate poor mental health outcomes related to EPL [2]. Specifically, it remains unknown if certain factors, such as the practice of self-compassion or the use of a support person might offset the emotional challenges that commonly follow an EPL.

The involvement of a non-clinical emotional and informational support person—or doula—during labor is associated with improved maternal and neonatal health outcomes, reduced incidence of perinatal medical complications, and decreased socioeconomic disparities in perinatal outcomes [3]. Positive patient experiences has been demonstrated when a trained support person is utilized during abortion procedures [4]. Doulas promote improved patient outcomes in part by supporting patients in pain management, helping patients to develop self-compassion, advocating for patients, and teaching patients salient coping strategies—such as breathing techniques and positive self-talk—both in the period before and after gynecologic procedures [3].

The utility and possible benefit of a peer emotional support person during management of EPL remains unexplored. One role for this potential support person could include providing guidance on self-compassion practices. Individuals with higher baseline self-compassion display a decreased likelihood of developing mood disorders postpartum, and that self-compassion interventions can offset the possibility of developing a mood disorder in the postpartum period [5]; however, self-compassion has not been explored within the context of EPL [6]. If practicing self-compassion is associated with a decreased incidence of poor mental health and maladaptive coping mechanisms following EPL, then one possible intervention for patients with EPLs could be a peer support person, trained in self-compassion practices. Additionally, there is a need for more general information on the unique emotional and/or informational support patients need to navigate their EPLs, how this support can best be provided, and if this type of support results in improved clinical outcomes and patient perception of their EPL experience. Such information could inform a potential EPL peer support program.

This study explores what types of support patients experiencing EPL feel they need and would benefit from, assesses patient interest in peer EPL support, and determines whether certain factors—such as perceived self-compassion—are important in processing EPL. The results of this study could inform the potential role that peer EPL support person could fill as an intervention for EPLs in the future.

## Methods

### Study design

We conducted a qualitative study utilizing semi-structured interviews. The interviews included questions exploring interest in a peer EPL support person for management of EPLs, perceived support needs of patients undergoing EPL based on personal experience, and specific questions about perceived self-compassion and the use of self-compassion practices. Prior to the interview, patients completed a pre-interview survey on RedCAP, a secure, web-based application designed to support data capture for research studies [7]. The survey contained demographic questions about relevant topics such as insurance status, partnered status, and self-identified race and ethnicity. The study was approved by our institution’s Human Research Protection Program (HRPP). All participants provided verbal informed consent to participate.

### Sampling and eligibility criteria

We recruited English-speaking women between the ages of 18 and 50 years-old who experienced EPL within the two years prior to study enrollment. Participants were recruited from a social media post on the Empty Cradle Facebook group.

### Interviewing and data collection

Patients participated in an individual 30-minute interview via videoconferencing. We utilized a semi-structured guide with open-ended questions that were designed to encourage discussion of participants’ EPL experiences, as well as concerns or suggestions for a possible peer EPL doula support program (see Appendix 1). Our interview questions were drafted using principles of patient-centered care and trauma-informed care in order to center the narrative of the interviewee and help offset the potential emotional challenges of discussing a sensitive topic [8, 9]. Interviews were video/audio recorded and transcribed. If a participant refused audio recording, then detailed notes were recorded instead at the time of the interview. To ensure accuracy of commentary, we reviewed the recordings and compared them to the call transcripts. All recordings were transcribed and coded. Participants were compensated with a $20 electronic gift card.

### Analysis

Dedoose was used to code the qualitative data via inductive and deductive content analysis [10]. Specifically, after the interviews were transcribed, two research team members read the transcripts and associated field notes. They familiarized themselves with the narratives and created codes to guide theme development. A codebook was developed based on recurring motifs. Two coders coded and used content analysis to analyze the interviews. Coding discrepancies were reviewed to reach consensus and ensure inter-coder reliability. Through iterative reflection, the study team members developed a list of emergent key themes and sub-themes seen across individual narratives and relevant codes. The coding and identification of themes was then reviewed with a third research team member. Themes were consolidated to achieve consensus, and the team developed a final interpretation of the data that accurately represented the interviews. We continued to collect interview data until thematic saturation was achieved.

## Results

### Study participants

Between May and August 2022, we recruited 21 women aged 18–50 years old who experienced EPL in the past two years. Approximately 52% (n = 11) percent of participants reported expectant management of their EPL, 23.8% (n = 5) reported medication management, and nearly 24% (n = 5) reported undergoing a D&C procedure. 61.9% (n = 13) of participants had been pregnant before at least once prior to their EPL, and the remaining 38.1% (n = 8) were nulliparous. 57% (n = 12) of those interviewed reported that their pregnancy that resulted in EPL was intended, and 90.5% (n = 19) of participants stated that their pregnancy resulting in EPL had been desired. Participant characteristics, including demographic information, are further summarized in Table 1.

**Table 1:** Demographic Characteristics of Participants in a Qualitative Study on Peer Support for EPL Patients (n = 21)

**Race**	
White	13 (61.90%)
Black	4 (19.05%)
Asian/Pacific Islander	1 (4.76%)
American Indian/Alaska Native	1 (4.76%)
More than one race	1 (4.76%)
Unknown/not reported	1 (4.76%)
**Age at time of EPL**	
Average	33.41
Standard deviation	6.34
**Ethnicity**	
Hispanic or Latino	3 (14.29%)
Not Hispanic or Latino	18 (85.71%)
**Annual Income**	
0 - $10,000	1 (4.76%)
$10,000–40,000	8 (38.10%)
$40,000–90,000	8 (38.10%)
$90,000 -170,000	3 (14.29%)
$170,000–215,000	1 (4.76%)
**Religion**	
Atheist	1 (4.76%)
Protestant	4 (19.05%)
Jewish	1 (4.76%)
Catholic	6 (28.57%)
Unaffiliated	14 (66.67%)
Buddhist	1 (4.76%)
**Insurance Coverage**	
Public	6 (28.57%)
Private/Commercial	14 (66.67%)
Uninsured	1 (4.76%)
**Means of payment for EPL**	
Patient’s insurance	14 (66.67%)
Partner’s insurance	2 (9.52%)
Out of pocket	5 (23.81%)
**EPL management**	
Expectant management	11 (52.38%)
Medication management	5 (23.81%)
Dilation and curettage (D&C)	5 (23.81%)
**Location of EPL care**	
Emergency Room	8 (38.10%)
OB-GYN office	10 (47.62%)
PCP office	3 (14.29%)
Other	10 (47.62%)
**Setting of EPL care**	
Community health center	9 (42.86%)
Academic institution	3 (14.29%)
Planned Parenthood	1 (4.76%)
Private practice	4 (19.05%)
Other	4 (19.05%)
**Pregnant before EPL**	
Yes	13 (61.90%)
No	8 (38.10%)
**Past pregnancy result**	
Living child	6 (28.57%)
EPL/miscarriage	6 (28.57%)
Abortion	4 (19.05%)
Other	0 (0%)
**Pregnancy that resulted in EPL intended?**	
Intended	12 (57.14%)
Unintended	9 (42.86%)
**Pregnancy that resulted in EPL desired?**	
Desired	19 (90.48%)
Undesired	2 (9.52%)
**Psychiatric diagnosis prior to EPL**	
Yes	8 (38.10%)
No	13 (61.90%)
**New psychiatric diagnosis since EPL**	
Yes	3 (14.29%)
No	18 (85.71%)

### Themes

The following five themes emerged from the data: [1] therapy and in-person support groups are helpful when experiencing EPL but are often inaccessible; [2] social media support groups are initially advantageous for creating a sense of solidarity, but in the long term can be triggering; [3] support from a peer who has also experienced EPL is uniquely valuable; [4] developing self-compassion is important in emotionally coping with EPL; and [5] there is a demand for emotional and informational support following EPL, which could be provided by a peer EPL support person .

**Theme 1: Therapy and in-person support groups are helpful and highly requested when experiencing EPL but are sometimes financially and geographically inaccessible.** Participants reported the use of therapy or support groups as essential in coping with their loss.

*I’m glad I had a therapist already, who’s been my therapist for the last two years…She had told me that she thought that I was starting to have postpartum depression.* − 25, Black/African American, public insurance, D&C.

*I feel like therapy has been the most helpful, because it’s like a time where I’m, like, forced to think about the grief process.* —39, Hispanic/Latine, private insurance, D&C.

*Going to the support groups gives hope, because I’m talking to people that are going through it, or have been through it. And it’s just like a really different level of comfort.* − 25, white, private insurance, expectant management.

However, the majority of participants wanted access to therapy or in-person support groups, but faced prohibitively long wait times, financial barriers, or logistical obstacles.

*I think that even one [therapy] session was super helpful, but like as I mentioned, it took almost like three months for a session to start. And I think just the wait times, the waitlists, for a lot of these services are really, really long, but when you’re going through your miscarriage, I think you need that support at that time. And it’s still nice to get it later on. But I think for me, the hardest month was when I was miscarrying.* − 38, American Indian/Alaska Native, private insurance, medication management.

*Yeah, and so when you find [a therapist] and you find out it’s all cash, out of pocket, and then they want like $500 for the first [visit], and then, like, you know, $350 for anything after that, and it’s only 45 min, you’re like, “That is insane!” So then it’s like one of those things that it’s like you put yourself and your healing on the back burner, because…in my situation, you know, I have my stepson, who needs to be fed, clothed, you know, all these things, and money needs to go towards that.* —25, white, private insurance, D&C.

Difficulty finding an in-network or affordable mental health clinician who was accepting new patients remained challenging across the board, suggesting that even the insured are underinsured with respect to mental healthcare. The utility of therapy was further constrained by therapists that did not specialize in grief, loss, or bereavement, or who were generally not well-versed in EPL.

*She [the therapist] said, “Yes, I have experience with grief and miscarriage.” But then anytime I brought up miscarriage in our conversations, it would get sidestepped or passed along.*

–36, white, private insurance, medication management.

*There are so many therapists and counselors and all of that out there, but the ones who specialize in child loss specifically are non-existent…People can say what they want, but losing a child is different than losing a parent. It’s different than losing an aunt, an uncle, a friend. It’s like this was like a part of you. This is something you grew.* − 25, white, private insurance, D&C.

**Theme 2: Social media support groups are initially advantageous for creating a sense of solidarity, but in the long term can be triggering.** When first joining online support groups, people primarily reported a sense of relief and solidarity in being connected to others who were also grieving.

*I usually don’t get too into Facebook groups, but…—it’s like “this is how it happened to me too” or even somebody just saying, “I’m sorry that you’re experiencing that,” or “You do have support; you’re not alone.”* − 40, white, private insurance, expectant management.

*Having so many people who are also… struggling with loss and going through the same experiences and worse, like, having their presence known makes it much more tolerable because then instead of just like scrolling and seeing baby after baby after baby, I just don’t feel as alone.* − 24, white, public insurance, expectant management.

However, over time, engagement with these groups could prolong the grieving process by triggering individuals with new stories of loss.

*I did join a couple of Facebook support groups. I recently snoozed them, just because it’s a large group of women who are constantly posting about their losses and just what a hard time they’re having, so I just felt like a vortex of me just getting dragged down, and not being supported. I just…I needed some distance. So you’re inundated with so much, especially since we’re hoping to try again soon, and I just don’t wanna be wrapped up in the anxiety of constant loss.* − 39, Hispanic/Latina, private insurance, D&C.

*[The social media group] was helpful for the beginning, but, like I said, the negativity that some people were sharing was a little too much for me.* − 35, Black or African American, public insurance, D&C.

Overall, engaging with social media seemed to provide an initial sense of connectedness to other people with similar experiences, but over the long term proved to be triggering as there was rapid group membership turnover and constant re-traumatization as new stories of loss were shared daily. This perhaps points to the benefits that could be gleaned from a more individualized approach rather than a large group platform.

**Theme 3: Support from a peer who has also experienced EPL is uniquely valuable due to a shared lived experience.** Many participants stated that they felt most comforted by other people who had experienced EPL due to a shared sense of understanding.

*…the only way that I felt comforted was if it was coming from somebody else who knew that pain. Because, I mean, people can have sympathy, but it just wasn’t the same coming from anyone else. But like the minute somebody told me, like, “I’ve also had a miscarriage. I lost a baby,” it gave a feeling of comfort that I couldn’t get anywhere else.* − 24, white, public insurance, expectant management.

*Instead of me having to try to pinpoint what it is, they [people who have had miscarriages]’ve experienced the same thing in their way, and so they can understand more of what it is that I am going through.* − 33, white, private insurance, D&C.

*There are people that kind of acknowledge that it happened, but then they forget that it’s not just like a “get over in a day” thing. She’s a friend [who has had a miscarriage] that recognizes, like, this is a process, it’s gonna take time to get over. And so that’s definitely there with her because she’s gone through that. So she sees it’s not just one day. It’s every day that you’re trying to push forward and like kind of move on. But yeah, I would say there’s something very different about those that have been through and understand and have felt that hurt, and they know what I’m feeling and what I need.* − 35, white, private insurance, expectant management.

*People who’ve had miscarriages and stuff they kind of understand what it’s like, and everything, and they know how to calm me down.* − 23, white, public insurance, expectant management.

Many participants reported that interfacing with a peer who had also experienced EPL helped them to feel less alone, to normalize conversation about grief and EPL, and to destigmatize negative feelings they held about their EPL. This in turn facilitated the development of self-compassion.

**Theme 4: Developing self-compassion is important in emotionally coping with EPL because it is required for overcoming feelings of shame, guilt, and self-blame.** Many participants stated that learning to practicing self-compassion, acknowledging the validity of their own emotions, and overcoming feelings of self-blame was pivotal in processing their EPL.

*Yeah, so I think I finally got to the point when like the negative thoughts would start rising like, “It’s your fault. It’s your fault. You should feel guilty,” and then being able to try to combat it. Like “No, no, you’re not—it’s not your fault. Your babies will always know that you love them, and that you wanted them and you know you wish…that they would have made it.”*

–33, Black/African American, private insurance, expectant management.

*Whenever you have feelings, you know you kind of honor those feelings and don’t try to suppress it. And when you are happy, you know, acknowledge that also. Overall, just kind of riding the waves.”* − 33, Black/African American, private insurance, expectant management.

*I do realize that it’s not my fault, but I had to like keep fighting those thoughts back and be kind to myself. Sometimes it makes me feel like…I feel as a mother, my body failed my babies. But I have to keep in mind…everything happens for a reason…I feel like it’s finally catapulting me into a new area of life, like I’m finding inspiration in the pain.* − 35, Black/African American, public insurance, D&C.

Learning to identify and fully emote their feelings helped individuals process their loss and any associated guilt. Over time, learning to acknowledge these emotions led to the development of self-compassion.

**Theme 5: A peer EPL support person could provide experiential insight during the EPL, as well as emotional support to navigate grief in the aftermath.** Many people stated that they wanted more informational support about what to expect *during* their EPL experience.

*We talk about miscarriage very briefly in society or whatever, and it seems like, “Oh, you just bleed a little bit, and then like that’s it.” But it’s like you actually, like, you give birth and you haven’t done anything like that, depending on what point in your pregnancy you are. Like I had no idea. So just knowing like maybe what’s to come, or what could be to come, would have been helpful.* − 35, white, private insurance, expectant management.

*I would have wanted somebody who could like spell out what was gonna happen.* − 35, white, private insurance, expectant management.

*I don’t remember, but there were side effects. Your bladder is different. and I would be rushing to the bathroom, and I was so confused, and it took a lot of time to figure out why, and it’s because my body was going through postpartum, even though I didn’t have a baby so [it] would have been great to ask somebody and get a quick answer instead of trying to find out myself, and then go in circles. And, you know, is this accurate information? …,So to have access to somebody could answer questions as well as access to emotional support would have been would have been great.* − 36, white, private insurance, medication management.

*Yeah, because it was women who…knew exactly what I was going through, you know…even like getting the D&C that I needed was traumatic in and of itself as well. And so being able to—well, being able to talk to [people who had also experienced miscarriage] helped me decide what the next step was for what I was gonna do to try to remove the dead tissue, you know. Whether it was gonna be like a natural process, or the pills, or the D&C. So it was helpful, because they had already gone through it, you know.* − 39, Hispanic/Latine, private insurance, D&C.

*And so having a support person that’s been there to be like, “Oh, yeah, that happened to me too,” or “Oh, yeah, I don’t know if that is normal, and I would get that checked out.” You know, something like that. I kind of overreacted about what was happening and caused more stress on myself than I needed to have.* − 35, white, private insurance, expectant management.

Following their EPL experience or procedure, people’s initial interest in informational support shifted to include an additional need for more long-term emotional support. Participants specifically expressed interest in receiving this type of support from a peer.

*I feel like if I had someone there that I could express my grief with, that I didn’t feel would be judgmental or feel…make me feel awkward, and I could just share openly, and just get it all out, that it would have made it that much better.* − 35, Black/African American, public insurance, D&C.

*You know, having that peer person, that support person…if it’s somebody who has gone through it, it might even be more beneficial because you go into this thing so blind like, “Okay, well, is what I’m feeling more…is it normal to be angry? Is it normal to cry? Like, why am I laughing? And then crying and then like hysterically laughing again?”* − 25, white, private insurance, D&C.

In summary, most individuals wanted access to informational support at the time of their EPL, while there was a pivot to a demand for emotional support following their EPL. Knowledge about the relative demand of different types of support relative to the timing of EPL can help tailor peer support person interventions moving forward.

## Discussion

Several participants highlighted the utility of in-person support groups and therapy services as useful in processing their EPL. However, most participants found these options to be geographically, financially, or logistically inaccessible, highlighting the need for more attainable support systems. In general, this is reflective of the national demand for mental healthcare clinicians that has only increased during the Covid-19 pandemic [11, 12]. Once individuals were able to find a clinician, many reported that they felt that the potential benefit of therapy was offset by therapists who were not well-versed in EPL and did not have specific expertise supporting individuals through pregnancy loss and bereavement. This finding is consistent with existing research that demonstrates the importance of access to specific, sensitive, EPL-focused mental health care following EPL [13]. In cases where participants were unable to access a mental healthcare clinician, many found that the use of other support tools—including developing personalized rituals and symbols or engaging with new hobbies—helped them to process and cope with their EPL. Importantly, though studies have underscored the importance of ritual in processing pregnancy loss, studies also suggest that patients can benefit from the combination of personalized rituals paired with support groups and therapy [14, 15]. Patients may therefore benefit more from an approach that integrates both.

Another common means of support that we identified was social media, especially the use of Instagram and Facebook support groups. Social media is a well-documented vehicle for connecting individuals and facilitating conversations about grief during healthcare-related crises, including perinatal loss [16-18]. Interestingly, our study revealed a double-edged aspect of using social media support groups as a coping tool in the context of EPL: while participants often commented on the initially positive feelings associated with identifying other people with a shared experience, many participants stated that over time, participation in the group lead to feelings of despair, re-traumatization, and depression. These findings are consistent with studies investigating the use of social media support groups for stillbirth and perinatal loss, which found that prolonged engagement with these support groups could sometimes amplify the intensity of feelings of grief [18]. When individuals reported that they found social media helpful, it was usually in the context of being connected with people who have experienced similar trauma; being connected to an individual peer EPL support person could perhaps also provide this sense of connection while simultaneously avoiding the triggers of being constantly exposed to new people’s EPL stories in a social media group setting with high turnover.

When reflecting on helpful in-person means of support, participants repeatedly emphasized the utility of being connected to other people who had experienced miscarriage. The main reasons for this seemed to be an unspoken sense of common understanding and solidarity that could not be gleaned from others, despite their well-meaning behavior and attempts to provide comfort. It is also possible that anger deflected towards others, the perception of being pitied, and the possibility of communicational misunderstanding are all minimized when receiving support from someone else who has also experienced EPL. Since the majority of participants endorsed support from a peer, this underscores the potential benefit of a pilot peer support EPL program to connect individuals with shared lived experience.

In reflecting on the benefits of being connected to peers who had also experienced EPL, a recurring concept was the utility of the peer in facilitating self-compassion. Self-compassion has been highlighted in many studies as an effective tool in coping with psychosocial disturbances, including various traumas [19]. Self-compassion—as defined by Neff, the creator of the self-compassion psychological framework—includes three main components: self-kindness instead of self-judgement when confronted with pain and adversity; conceptualizing suffering as a shared human experience instead of feeling isolated by it; and practicing mindfulness in the face of distressing feelings as opposed to over-identifying with them [20]. Listening to a peer process their own feelings associated with EPL often led to participants reminding the peer that they were not at fault for the outcome of their pregnancy. This in turn helped participants overcome their own misplaced feelings of guilt and self-blame, fostering self-kindness and self-understanding instead.

Another novel finding of this study was that participants indicated that at the time of their EPL, they wanted improved access to informational support, while in the long-term following their EPL, there was a greater demand for emotional support. Short term informational support requested included the ability to call or text someone with questions about the EPL experience and what to expect in real time; long term emotional support was described as having someone to call or text to process feelings of grief. Most interviewees felt that peers who had also experienced EPL may be uniquely poised to provide this type of support, and that it would be most helpful to be able to virtually access a peer support person via call or text as needed.

Support persons are well characterized as an intervention in other obstetric contexts [21]. However, this study is one of the first to better characterize the specific type of support patients experiencing EPL may need. Additionally, studies that have provided a support person intervention have provided only point of care clinical support and a one month follow up assessment [21, 22]. Our study demonstrates that many EPL patients have a desire for more long-term support following their EPL. A peer EPL support person could feasibly provide this unmet need for longer term support. The creation of a pilot peer EPL peer support program will help us to better understand the benefits, shortcomings, and outcomes of using this type of intervention.

A strength of this study was utilizing a qualitative research design, which provides in-depth insight into the current landscape of EPL support. There was also a diverse sample with different geographic locations and type of management. In addition, the participants were ethnically diverse. However certain groups—especially Latine participants—were underrepresented, due to exclusion of Spanish speakers.

There are several limitations to this study. Although we achieved thematic saturation, our sample size was small, as it proved challenging to recruit participants willing to talk about a sensitive, sometimes stigmatized, and deeply personal topic. In addition, participants were recruited primarily from social media, which could have resulted in a selection bias. The results may be skewed towards individuals who were struggling to cope with EPL and actively sought out support from online forums. In fact, our recruitment approach itself may have over highlighted the usefulness of social media support groups which was our Theme 2. A different recruitment approach may not have yielded this theme. In addition, the majority of participants reported desired pregnancies, which may have contributed to the challenges they experienced in processing their EPLs and thus biased our findings. However, asking participants to label their pregnancy within the binary of “desired” or “undesired” may oversimplify the complexity of emotions associated with processing a pregnancy. Furthermore, since almost all recruitment was exclusively via social media EPL groups, the results may not be generalizable to those that do not routinely use technology.

## Conclusions

The results of this study demonstrate a need for more support for EPL patients, and that having access to a peer EPL support program is one possible modality for providing this support. Notably, our study highlights that many people who have experienced EPL feel that the support they receive from their peers is uniquely helpful, perhaps underscoring the unique benefits of a peer support program. The findings of this study support the utility of a peer EPL support program. Patients desire access to a vetted individual who has a shared experience and can provide on call, point of care informational support as well as long-term emotional support. Further studies are required to determine the specific components of a peer EPL support program and to pilot such a program.

## Electronic supplementary material

Below is the link to the electronic supplementary material.

Supplementary Material 1

## Data Availability

The datasets analyzed during the current study are available from the corresponding author upon reasonable request.

## List of abbreviations

EPL: Early pregnancy loss
D&C: Dilation and curettage
HRPP: Human Research Protection Program
UCSD: University of California, San Diego
