# Supplementary material for: Interest in peer support persons among patients experiencing early pregnancy loss
Source: BMC Pregnancy Childbirth. 2023 Jul 11;23:506. doi: 10.1186/s12884-023-05816-x (PMC10334662; doi:10.1186/s12884-023-05816-x)
Supplement: Supplementary file 1 — Supplementary Material 1 [file 12884_2023_5816_MOESM1_ESM.docx]

**Appendix 1: Interview Guide of a Qualitative Study on Peer Support for EPL Patients** (page 5, line 10)

*Support system:*

1. Relationship status: Were you partnered or single at the time of your EPL?

2. Living circumstances: With whom (if anyone) were you cohabitating at the time of your EPL? (i.e. family, partner, spouse, friends, roommates, etc.)

3. Interpersonal support: What types of interpersonal support did you use during and after your EPL? (i.e. family, spousal, partner, friends, social media, online support groups, etc.)

4. Support tools: What kinds of support tools did you use during and after your EPL? (i.e. journaling, art, exercise, meditation, therapy, etc.)

5. Accessibility: How accessible were the types of support you used? Was accessing any type of support especially challenging, cost-prohibitive, or unavailable?

6. Needs: What type of support do you feel was indispensable/essential to navigating your EPL?

*Self-compassion and baseline coping mechanisms:*

7. Impact: In what ways did your EPL impact you (i.e. emotionally, physically, interpersonally, professionally, etc.)?

8. Baseline perception of self-compassion: How would you describe your baseline perception of self-compassion?

9. Post-EPL self compassion: Did your EPL experience affect your sense of self- compassion? What role did self-compassion play in navigating your EPL experience?

*EPL doula interest:*

10. Interest: Would you have been interested in having access to a peer support person or doula during your EPL? Does this sound like an intervention that you might recommend to other EPL patients? What is the likelihood that you would request or recommend a peer support person as an EPL intervention?

11.Peer component: Do you think that a peer (someone who has also experienced an EPL) is uniquely positioned to support other EPL patients? Would you prefer to have a peer support you during an EPL than another person (i.e. a friend, partner, etc.)?

12.Self-compassion aspect: Should the hypothetical peer doula program involve self-compassion education to help address the psychological impact of EPL?

13. Concerns: What concerns do you have about the potential use of a peer support person as an EPL intervention? Is there any reason you might be hesitant to recommend this resource?
